# Supplementary material for: Postoperative Karnofsky performance status prediction in patients with IDH wild-type glioblastoma: A multimodal approach integrating clinical and deep imaging features
Source: PLoS One. 2024 Nov 11;19(11):e0303002. doi: 10.1371/journal.pone.0303002 (PMC11554073; doi:10.1371/journal.pone.0303002)
Supplement: S1 Fig — (PDF) [file pone.0303002.s001.pdf]

S1 Fig. Participants flow

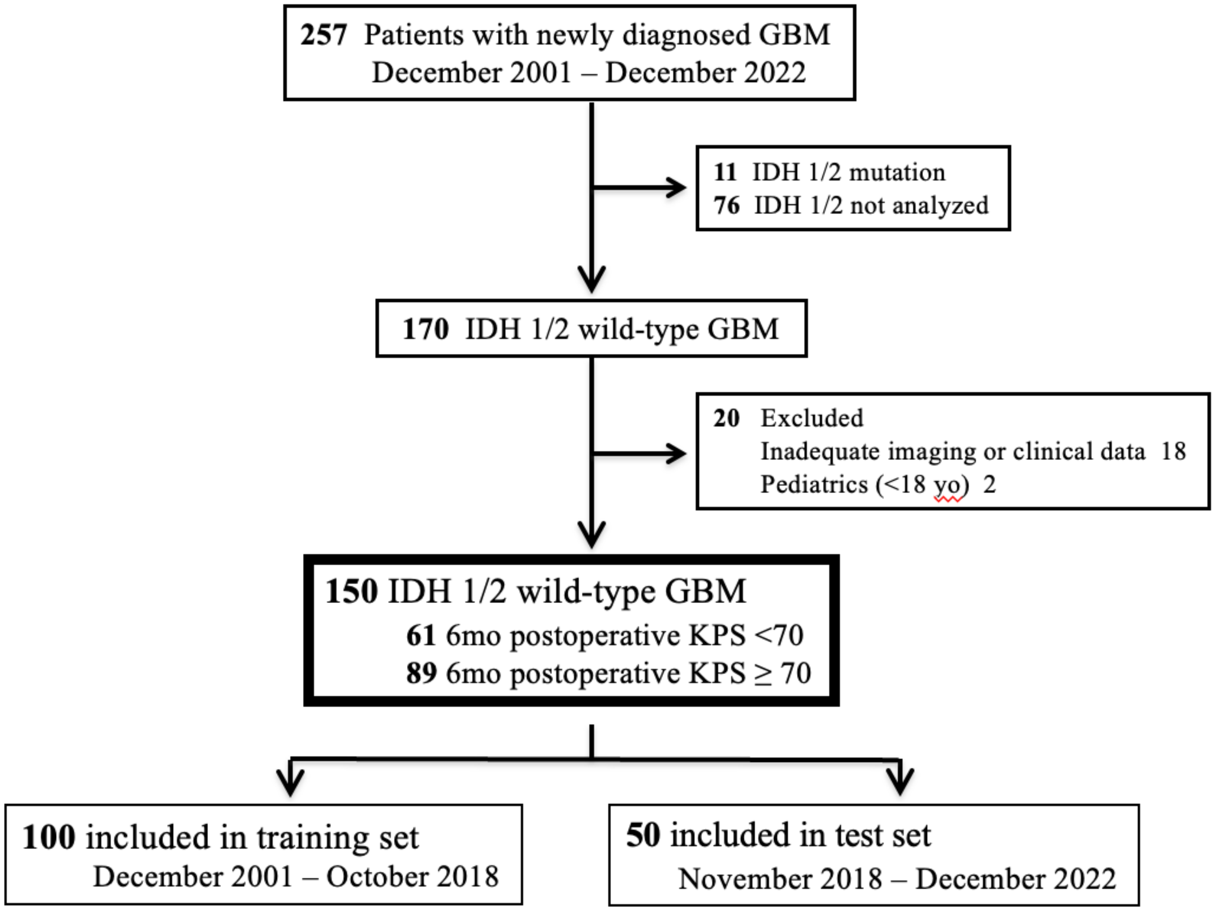

Flow scheme outlining the participants selection for this study.

GBM, glioblastoma. IDH 1/2, isocitrate dehydrogenase 1/2. KPS, Karnofsky performance status.
