# Supplementary material for: Postoperative Karnofsky performance status prediction in patients with IDH wild-type glioblastoma: A multimodal approach integrating clinical and deep imaging features
Source: PLoS One. 2024 Nov 11;19(11):e0303002. doi: 10.1371/journal.pone.0303002 (PMC11554073; doi:10.1371/journal.pone.0303002)
Supplement: S2 Fig — (PDF) [file pone.0303002.s002.pdf]

S2 Fig. The architecture of the variational autoencoder

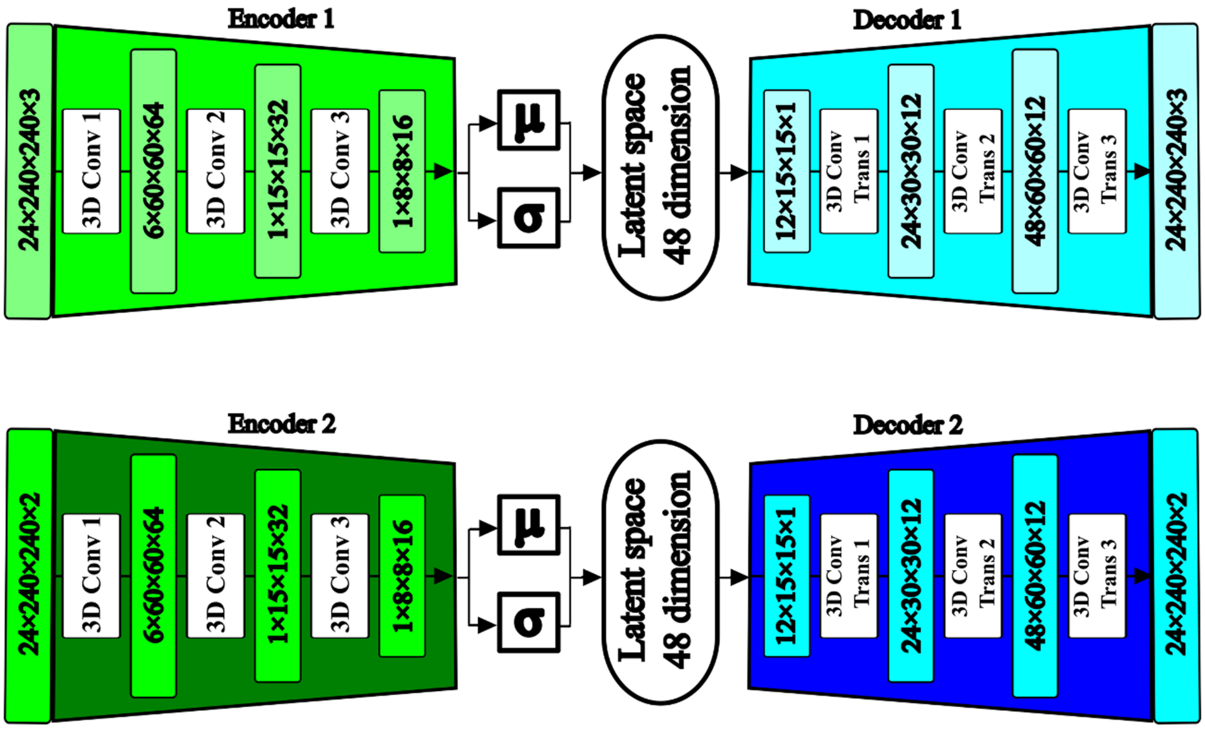

Encoder 1 comprised three layers of 3D convolutional blocks with 64, 32, and 16 learnable filters, each employing a kernel size of 3. The first and second convolutional blocks were followed by a rectified linear unit (ReLU), max pooling, and a dropout layer with a connection dropout probability of 0.5. The third convolutional block was followed by a flatten layer, a dense layer, and layers for calculating the mean and log variance, resulting in a 48-dimensional latent space. Decoder 1 utilized two fully-connected dense layers and three transposed convolutional layers to reconstruct the representations from the encoder into the original tumor lesion images.

Encoder 2 and Decoder 2 mirrored the structure of Encoder 1 and Decoder 1, respectively. The difference between Encoder 1 and Encoder 2 lay in their input and output configurations. Encoder 1 received images with three channels: black (blank), red (enhancing tumor and necrosis, cysts), and green (non-enhancing tumor and perifocal edema). Its corresponding output from Decoder 1

also featured three channels. In contrast, Encoder 2 processed input with two channels: black and gray (brain parenchyma). Similarly, Decoder 2 reconstructed output with two channels. The autoencoder was trained by minimizing both the reconstruction loss and the Kullback-Leibler divergence loss between the input and reconstructed signals.
