# Supplementary material for: Postoperative Karnofsky performance status prediction in patients with IDH wild-type glioblastoma: A multimodal approach integrating clinical and deep imaging features
Source: PLoS One. 2024 Nov 11;19(11):e0303002. doi: 10.1371/journal.pone.0303002 (PMC11554073; doi:10.1371/journal.pone.0303002)
Supplement: S3 Fig — (PDF) [file pone.0303002.s003.pdf]

S3 Fig. The KPS prediction model development using neural network

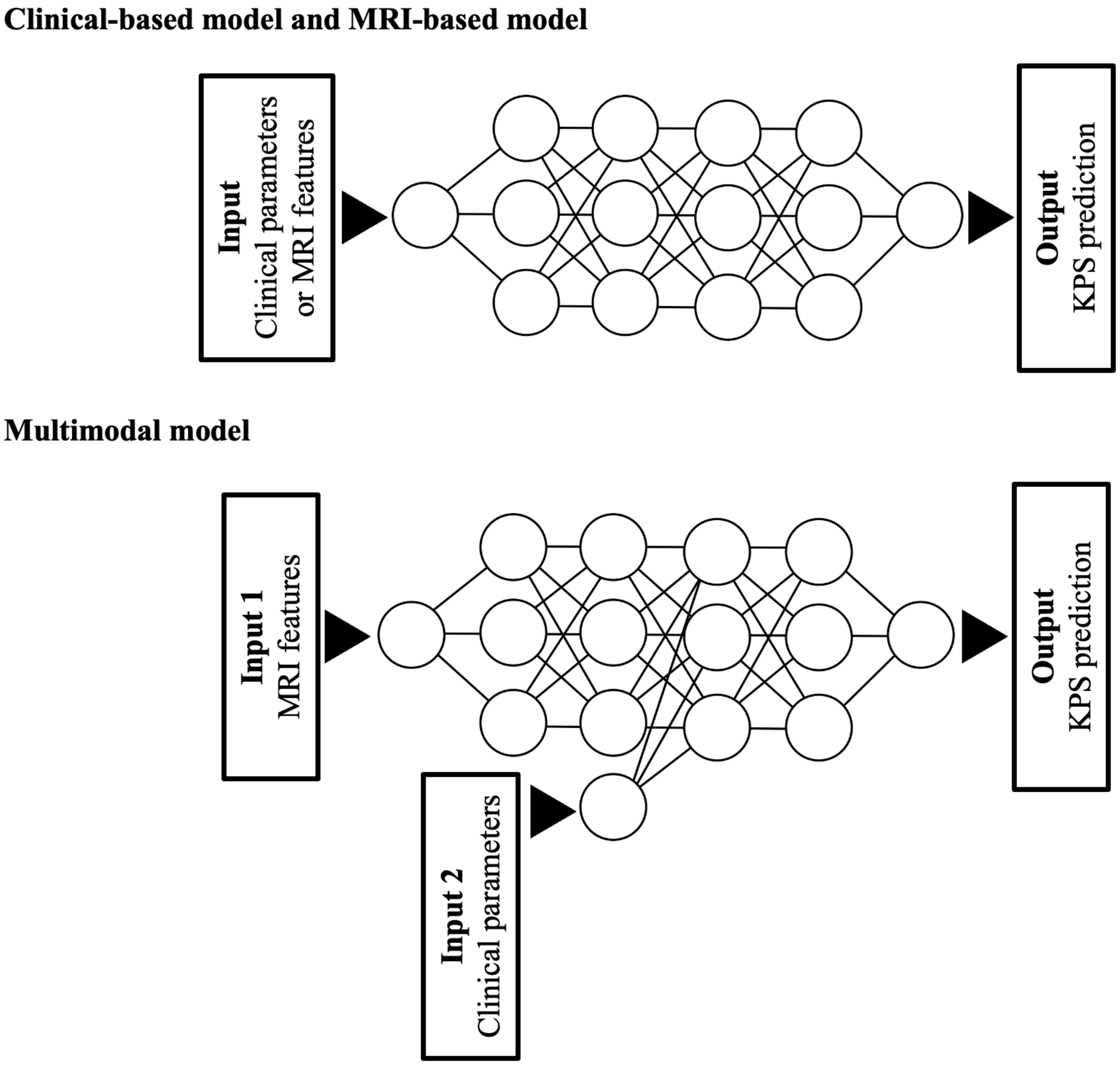

In the clinical-based model, clinical parameters are integrated into three dense layers equipped with ReLU activation and layer normalization. The final output layer comprises 2 neurons with a softmax activation function, which is ideal for KPS prediction.

In the MRI-based model, MRI features are incorporated into seven dense layers with ReLU activation and layer normalization. The final output layer consists of 2 neurons.

In the multimodal model, deep imaging features serve as the primary input and pass through three dense layers with ReLU activation and layer normalization. The output from these layers is then merged with the second input, which includes clinical features. This combined data proceeds through

an additional six dense layers followed by ReLU activation and layer normalization. The final output layer comprises 2 neurons.

To prevent overfitting, the network employs L2 regularization and employs the categorical cross-entropy loss function for training. The learning process is driven by the Adam optimizer with a learning rate of 0.0005.
