# Supplementary material for: Postoperative Karnofsky performance status prediction in patients with IDH wild-type glioblastoma: A multimodal approach integrating clinical and deep imaging features
Source: PLoS One. 2024 Nov 11;19(11):e0303002. doi: 10.1371/journal.pone.0303002 (PMC11554073; doi:10.1371/journal.pone.0303002)
Supplement: S4 Fig — (PDF) [file pone.0303002.s004.pdf]

S4 Fig. Comparison between the neural network and other machine learning algorithm

Mean score among 5-fold CV	RFC	XGB	LGB	NN
AUC	0.663 ± 0.020	0.705 ± 0.029	0.720 ± 0.23	**0.785 ± 0.051**
P value (vs NN)	**<0.001**	**<0.001**	**0.01**	**-**

※ Mean score ± Standard deviation

Mean score among 5-fold CV	RFC	XGB	LGB	NN
Accuracy	0.658 ± 0.019	0.654 ± 0.017	0.689 ± 0.027	0.728 ± 0.032
Specificity	0.931 ± 0.025	0.914 ± 0.025	0.812 ± 0.023	0.847 ± 0.057
Sensitivity	0.215 ± 0.027	0.234 ± 0.043	0.489 ± 0.044	0.529 ± 0.091
F1 score	0.307 ± 0.032	0.309 ± 0.047	0.539 ± 0.040	0.572 ± 0.066

※ Mean score ± Standard deviation

The performance metrics of each machine learning algorithm evaluated by 5-fol cross validation 10 times repeats. Data are shown in mean score ± standard deviation.

RFC, Random Forest Cassifier. XGB, XgBoost. LGB, Light Gradient Boosting Machine.
