# Supplementary material for: Postoperative Karnofsky performance status prediction in patients with IDH wild-type glioblastoma: A multimodal approach integrating clinical and deep imaging features
Source: PLoS One. 2024 Nov 11;19(11):e0303002. doi: 10.1371/journal.pone.0303002 (PMC11554073; doi:10.1371/journal.pone.0303002)
Supplement: Attachment — Submitted filename: Response_to_Reviewers.docx [file pone.0303002.s005.docx]

**Detailed point-by-point responses to the Reviewers**

October 3, 2024

Thank you very much for your invaluable advice. We want to thank the editor and reviewers for their effort and time in reviewing our manuscript. We have carefully considered each comment. We fundamentally agree with all the comments and have incorporated the suggestions into the revised manuscript. The responses to the reviewers and the changes made in the revised manuscript are shown below.

**Response to Reviewer #1**

**Comment 1:**

*" This is very well written paper and the rationale is well thought out. I enjoyed	reading this paper. I have only very minor comments. The paper would be	enhanced by 1) discussing the limitations of the methodology, 2) the	prospects for validation and	3) by comparing this analysis and its results with	other similar studies."*

**Author's response:**

We really appreciate your careful review and kind comments. We have revised the Discussion and Limitations sections according to your suggestions.

1) Discussing the limitations of the methodology

One of the main limitations in the development of our prediction model relates to the extraction of MRI features. Given the rapid advancements in deep learning architectures for medical image processing, this study employed only convolutional neural networks (CNNs) and variational autoencoders (VAEs). Incorporating more recent architectures like vision transformers can potentially enhance the performance of the KPS prediction model.

2) The prospects for validation

The nature of the retrospective-single center analysis leads to potential selection bias. Multicenter external test sets, based on a prospective design, are necessary to verify the generalizability of the model.

3) Comparing this analysis and its results with other similar studies

Research predicting the course of performance status in patients with malignant tumors is limited, in part due to the scarce availability of publicly accessible, precise clinical databases. However, in recent years, several studies have successfully employed machine learning approaches using clinical features to predict changes in performance status. These studies utilized only clinical features and developed unimodal models. As indicated in our study, a multimodal model that incorporates both clinical and imaging features can predict the performance status with significant accuracy in patients with malignant tumors, including glioblastoma.

**Changes to the text:**

“Multicenter external test sets, based on a prospective design, are necessary to verify the generalizability of the multimodal model.” (Limitations: page 18–19, lines 387–388)

“Given the rapid advancements in deep learning architectures for medical image processing, this study employed only convolutional neural networks and VAEs. Incorporating more recent architectures like vision transformers may enhance the performance of our KPS prediction model.” (Limitations: page 19, lines 390–393)

“However, in recent years, several studies have reported that machine learning approaches utilizing clinical features have successfully predicted changes in performance status. Using data from patients with glioblastoma, Della Pepa et al. developed a prediction model for KPS deterioration at 6 months postoperatively, achieving an AUC of 0.81 [10]. In another report, the prediction of poor performance status in patients with systemic cancer 6 months after diagnosis achieved an AUC of 0.807 [29]. These studies utilized only clinical features and developed unimodal models. As indicated in our study, a multimodal model that incorporates both clinical and imaging features can predict performance status with significantly greater accuracy in patients with malignant tumors, including glioblastoma.” (Discussion: page 18, lines 363–371)

**Response to Reviewer #2**

**Comment 1:**

*" Please consider revising the statement in line 67-69, “The 6-month	postoperative KPS was chosen for its clinical practicality, as it aligns with the	standard timeframe for evaluating disease progression after a typical course of	adjuvant treatments.” Most glioblastoma patients are in the middle of adjuvant	treatment. "*

**Author’s response:**

We are grateful for your thoughtful review. We fully agree that many patients are in the middle of adjuvant therapy at the 6-month postoperative mark in the standard protocol for glioblastoma. Our original statement was indeed misleading. We revised this sentence as follows.

**Changes to the text:**

“Commonly, the 6-month postoperative KPS score is used for evaluating disease progression in glioblastoma clinical trials [5].” (Introduction: page 4, lines 63–64)

**Comment 2:**

*" It would be important to address how the pre-surgical, intra-surgical variables	and other clinical factors were selected."*

**Author’s response:**

We appreciate your crucial comment. The pre-surgical, intra-surgical, and other clinical parameters used in our study are based on similar variables used by previous machine learning research on prediction models of glioblastoma prognosis [1,2]. These parameters are routinely assessed and available in clinical practice.

**Changes to the text:**

“According to previous research on machine-learning-based models predicting glioblastoma prognosis, the following 28 variables were incorporated as clinical parameters [10,11].” (Materials and methods: page 6, lines 115–116)

**Comment 3:**

*" It is unclear why patients were assigned into training and test sets by time range	instead of randomly. "*

**Author’s response:**

Thank you for your insightful comment. In studies with relatively small populations, the performance of a model on a test set can vary significantly with each random split, largely due to substantial changes in the distribution of the test set data [3]. Our study, with a cohort of 150 cases, may experience fluctuations in model performance that could either overestimate or underestimate the true performance, depending on the distribution of the test set in each random split. To mitigate this randomness and prevent the bias of arbitrarily selecting the test set through repeated random splits, we chose to split the cases based on the time of surgery into training and test sets in a 2:1 ratio. This method of splitting has been frequently employed in previous studies with similar cohort sizes to ensure reliability in the evaluation process [4,5].

**Changes to the text:**

“In studies with relatively small populations, the performance of a model on a test set can significantly vary with each random split due to substantial changes in the distribution of the test set data [7]. To mitigate the bias associated with arbitrarily selecting the test set through repeated random splits, we chose to divide the cases based on their time range into training and test sets in a 2:1 ratio, a method frequently employed in prior studies with similar cohort sizes [8,9].” (Materials and methods: page 6, lines 106–111)

**Comment 4:**

" Despite the robust MRI-based model building utilizing the VAE, the multimodal	model only improves the predictability slightly compared to clinical-only model."

**Author’s response:**

We appreciate your insightful comment. Indeed, the MRI-only model exhibited inferior performance compared to the clinical-only model in the training set (Figs 4A, B). Consequently, incorporating MRI features into the clinical variables slightly improved prediction performance relative to the clinical-only model (Fig 4E). Additionally, the grouped permutation feature importance analysis revealed that the top three variables contributing to the performance of the multimodal model were clinical features (Fig 4D). Although our findings suggest that imaging features have a lesser impact than clinical features on the performance of predictive models for glioblastoma patients, the integration of clinical parameters with MRI features consistently improved prediction accuracy. These results highlight the potential benefits of enhancing the clinical-based model with imaging features to improve prediction performance.

**Changes to the text:**

“Although MRI features had less impact on prediction performance than clinical features, we consistently observed an improvement in prediction performance when incorporating both clinical parameters and MRI features.” (Discussion: page 17, lines 339–342)

**Comment 5:**

*" It is unclear how could the KPS prediction model contribute to the	surgical planning, as the modeling requires pre- and post-operative MRIs."*

**Author's response**

We are grateful for your invaluable comment. We apologize for any ambiguity in our initial discussion. We anticipate a scenario where AI-generated postoperative images, rather than actual images, are integrated into a KPS prediction model for surgical planning. Recent research has successfully developed surgical resectability prediction models and generative models that produce high-quality images of both pre- and postoperative MRIs [6–8]. Integrating AI-generated postoperative images into a KPS prediction model may clarify optimal resection strategies and significantly enhance surgical simulations, thereby improving patient outcomes.

**Changes to the text:**

“The prediction of prognosis using pre- and postoperative MRI may also contribute to surgical planning when combined with generative artificial intelligence (AI) that synthesizes postoperative images.” (Discussion: page 18, lines 375–377)

“Furthermore, a recent study has demonstrated that generative AI models are capable of producing fine-quality images of pre- and postoperative brain tumors and normal parenchyma [32]. Integrating AI-generated postoperative images with a KPS prediction model might clarify optimal resection strategies and considerably enhance surgical simulations.” (Discussion: page 18, lines 380–383)

**References**

1.	Della Pepa GM, Caccavella VM, Menna G, Ius T, Auricchio AM, Chiesa S, et al. Machine learning–based prediction of 6-month postoperative Karnofsky performance status in patients with glioblastoma: capturing the real-life interaction of multiple clinical and oncologic factors. World Neurosurg. 2021;149: e866–e876. doi:10.1016/j.wneu.2021.01.082

2.	Della Pepa GM, Caccavella VM, Menna G, Ius T, Auricchio AM, Sabatino G, et al. Machine learning-based prediction of early recurrence in glioblastoma patients: a glance towards precision medicine. Neurosurgery. 2021;89: 873–883. doi:10.1093/neuros/nyab320

3.	Xu Y, Goodacre R. On splitting training and validation set: a comparative study of cross-validation, bootstrap and systematic sampling for estimating the generalization performance of supervised learning. J Anal Test. 2018;2: 249–262. doi:10.1007/s41664-018-0068-2

4.	Park JE, Kim HS, Lee J, Cheong EN, Shin I, Ahn SS, et al. Deep-learned time-signal intensity pattern analysis using an autoencoder captures magnetic resonance perfusion heterogeneity for brain tumor differentiation. Sci Rep. 2020;10: 1–11. doi:10.1038/s41598-020-78485-x

5.	Yun J, Yun S, Park JE, Cheong EN, Park SY, Kim N, et al. Deep learning of time-signal intensity curves from dynamic susceptibility contrast imaging enables tissue labeling and prediction of survival in glioblastoma. AJNR Am J Neuroradiol. 2023;44: 543–552. doi:10.3174/ajnr.A7853

6.	Miao X, Chen H, Tang M, Huang D, Gao T, Chen Y. Post-operative MRI synthesis from pre-operative MRI and post-operative CT using conditional GAN for the assessment of degree of resection. Displays. 2024;83: 102742. doi:10.1016/j.displa.2024.102742

7.	Marcus AP, Marcus HJ, Camp SJ, Nandi D, Kitchen N, Thorne L. Improved prediction of surgical resectability in patients with glioblastoma using an artificial neural network. Sci Rep. 2020;10: 1–9. doi:10.1038/s41598-020-62160-2

8.	Mukherkjee D, Saha P, Kaplun D, Sinitca A, Sarkar R. Brain tumor image generation using an aggregation of GAN models with style transfer. Sci Rep. 2022;12: 1–17. doi:10.1038/s41598-022-12646-y
